# The emerging role of exosomes in radiotherapy

**DOI:** 10.1186/s12964-022-00986-1

**Published:** 2022-10-31

**Authors:** Zhenyi Yang, Wen Zhong, Liang Yang, Ping Wen, Yixuan Luo, Chunli Wu

**Affiliations:** Fourth Affiliated Hospital of China Medical University, Liaoning, China

**Keywords:** Radiotherapy, Exosomes, Radioresistance, Bystander effect

## Abstract

Presently, more than half of cancer patients receive radiotherapy to cure localized cancer, palliate symptoms, or control the progression of cancer. However, radioresistance and radiation-induced bystander effects (RIBEs) are still challenging problems in cancer treatment. Exosomes, as a kind of extracellular vesicle, have a significant function in mediating and regulating intercellular signaling pathways. An increasing number of studies have shown that radiotherapy can increase exosome secretion and alter exosome cargo. Furthermore, radiation-induced exosomes are involved in the mechanism of radioresistance and RIBEs. Therefore, exosomes hold great promise for clinical application in radiotherapy. In this review, we not only focus on the influence of radiation on exosome biogenesis, secretion and cargoes but also on the mechanism of radiation-induced exosomes in radioresistance and RIBEs, which may expand our insight into the cooperative function of exosomes in radiotherapy.

**Video abstract**

**Supplementary Information:**

The online version contains supplementary material available at 10.1186/s12964-022-00986-1.

## Introduction

Radiotherapy has been applied as a mainstay treatment for cancer. More than half of cancer patients receive radiotherapy to cure localized cancer, palliate symptoms, or control the progression of cancer [1]. Radiotherapy an also be combined with surgery, chemotherapy or immunotherapy to achieve a better therapeutic effect. However, radioresistance and local relapse accompanied by metastasis remain challenges in the treatment of cancer patients. Although there are some clinical approaches, such as hyperfractionation and higher doses [2], to counteract radioresistance, it is still a seemingly unsolvable problem that reduces the curative effect of radiotherapy. We have found that exosomes, as a kind of extracellular vesicle (EV), seem to play a very important role in the mechanism of radioresistance. Radiotherapy, as a kind of environmental stress, tends to disrupt the homeostasis of the tumor microenvironment. Exosome secretion was initially proposed to maintain cellular homeostasis. Radiation induced lethal cytotoxicity and tumor cell apoptosis chiefly by breaking double-stranded DNA [3]. Radiation-induced exosomes could irreparably transport radiation-damaged DNA from irradiated cells to the extracellular environment to stop the activation of programmed cell death. Therefore, exosomes hold great promise for clinical application in reducing radioresistance.

Radiotherapy can induce bystander effects, not only on nonirradiated tumor cells leading to tumor cell death but also on normal cells and tissue leading to radiation injury [4]. Exosomes can transmit the radiation-induced bystander effect (RIBE) by using miRNAs as effector molecules [5, 6]. Therefore, exosomes may be a potential therapeutic target to kill nonirradiated tumor cells and protect nonirradiated normal cells.

In this review, we describe the influence of radiotherapy on exosome biogenesis and secretion and cargoes, placing a particular focus on the role of radiation-induced exosomes in radioresistance and RIBEs. These advances expand our insight into the role of exosomes in radiotherapy and new treatments of cancer.

## The influence of radiotherapy on exosome biogenesis and secretion

**Fig. 1:**
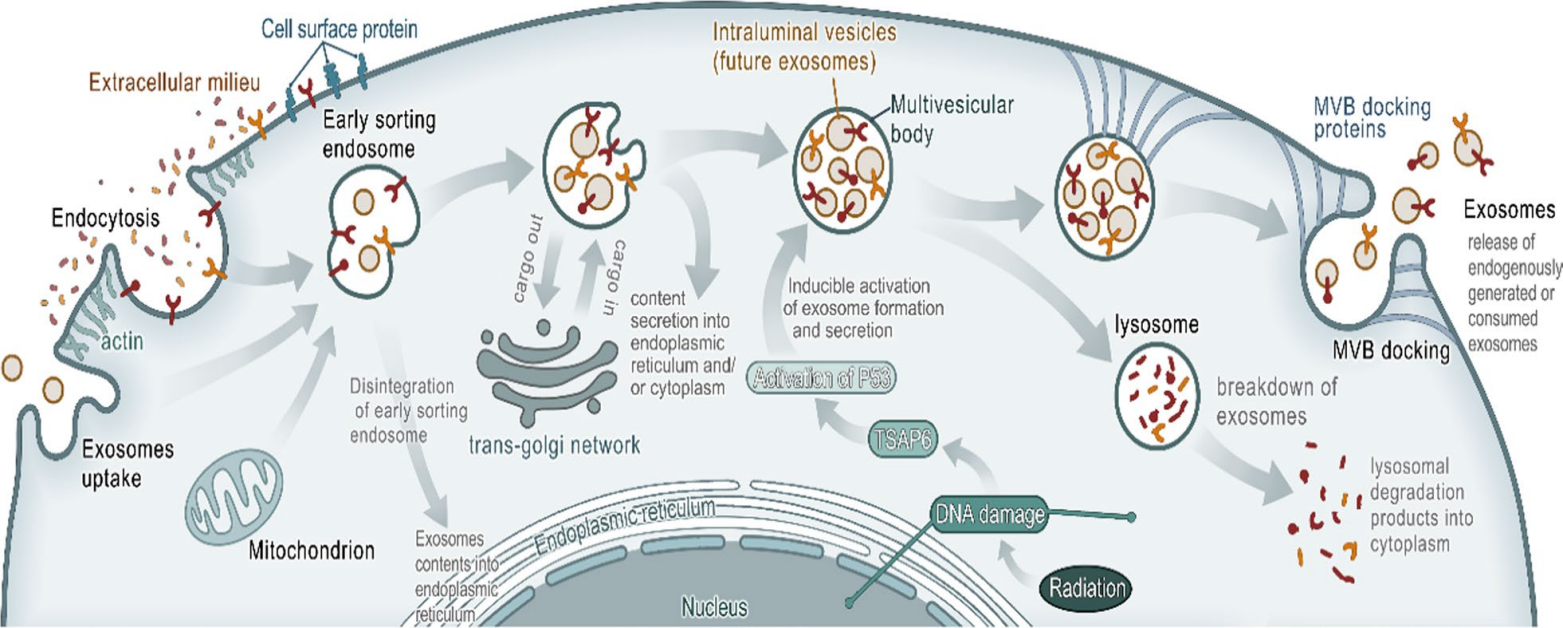
Exosome biogenesis and secretion. Fluid and extracellular constituents such as proteins, lipids, and small molecules can enter cells, along with cell surface proteins, through endocytosis and plasma membrane invagination. Then, the cup-shaped structure enters the de novo formation of an early-sorting endosome (ESE), including processes such as exchanging cargoes with the trans-Golgi network and endoplasmic reticulum and taking in some cargoes from mitochondria or merging with a pre-existing ESE. Next, the ESE turns into a late-sorting endosome (LSE). Then, the late-sorting endosomal membrane is invaginated to generate intraluminal vesicles (ILVs) and to further modify the cargoes of future exosomes with cytoplasmic constituents. After the defined collection of ILVs (future exosomes), LSEs are turned into MVBs. Radiation can cause DNA single-strand breaks (SSBs) and double-strand breaks (DSBs) in tumor cells. After DNA damage, tumor suppressor-activated pathway 6 (TSAP6) is activated. Then the p53 protein is activated to become a transcription factor and involve in the formation of exosomes. MVBs can fuse with autophagosomes, and ultimately the contents can undergo degradation in the lysosomes. MVBs can also be transported to the plasma membrane through the cytoskeletal and microtubule network of the cell and dock on the luminal side of the plasma membrane with the help of MVB-docking proteins. Exocytosis follows and results in the release of the exosomes

Exosomes are generated by outward budding of the endosome. Exosomes mainly include cargoes such as DNA, miRNA, mRNA, proteins and lipids, which have a significant function in mediating and regulating intercellular signaling pathways [7, 8]. The biogenesis of exosomes includes the formation of endosomes, the formation of multivesicular bodies (MVBs), the formation of intraluminal vesicles (ILVs) and then the secretion or degradation of exosomes (Fig. 1) [7–9]. At the onset, a cup-shaped structure containing cell-surface proteins and soluble proteins related to the extracellular milieu is formed by the invagination of the plasma membrane. Then, the cup-shaped structure enters the de novo formation of an early-sorting endosome (ESE), including processes such as exchanging cargoes with the trans-Golgi network and endoplasmic reticulum and taking in some cargoes from mitochondria or merging with a pre-existing ESE. Next, the ESE turns into a late-sorting endosome (LSE). Then, the late-sorting endosomal membrane is invaginated to generate intraluminal vesicles (ILVs) and to further modify the cargoes of future exosomes with cytoplasmic constituents. After the defined collection of ILVs (future exosomes), LSEs are turned into MVBs. Finally, the MVBs fuse with the plasma membrane to release the contained ILVs as exosomes with the help of MVB docking proteins or combined with lysosomes to enter the process of degradation. This model of exosome biogenesis, primarily enlightened by a study [10] on the vesicular secretion of TfR in maturing reticulocytes, is generally accepted by researchers and has gradually become the standard model of exosome biogenesis. However, there is still much evidence showing that exosomes can directly bud from the plasma membrane [11]. Recently, some researchers [8] hypothesized that exosomes bud from the plasma membrane and the endosomal membrane, and the reason for the controversy is observational bias.

Once the MVBs escape degradation, secretion begins. First, MVBs are transported to the plasma membrane by the cytoskeleton (microtubules and actin) [12] with the help of Rab GTPases, including Rab27b [13–16]. Then, the MVBs are docked at the luminal side of the plasma membrane with the help of Rab27a/b [13–16] and MVB-docking proteins. Next, MVBs fuse with the plasma membrane via soluble N-ethylmaleimide-sensitive fusion attachment protein receptors (SNAREs), such as synaptosome-associated protein 23 (SNAP-23) and synaptotagmin family members [17]. Finally, the exosomes are released into the extracellular environment with the help of exocytosis.

The complicated mechanism of exosome biogenesis and secretion is modulated by various factors and stress. Radiation, as a kind of external stress, can affect exosome biogenesis and secretion by the DNA damage response. Radiation therapy [3] can cause DNA single-strand breaks (SSBs) and double-strand breaks (DSBs). After DNA damage, the p53 protein, which can respond to a wide variety of stress signals, is activated to become a transcription factor and enhances or represses the transcription of a number of genes, which ultimately induce cell cycle arrest, senescence or apoptosis [18, 19]. Part of the p53 response to stress produces can secrete proteins that can communicate with adjacent cells, which is also called the “bystander effect” [20–22]. This process is classically mediated by a signal sequence at the NH2 terminus of a secreted protein that directs the protein through the endoplasmic reticulum/Golgi pathway followed by transport to the plasma membrane and release of the protein into the extracellular environment via exosomes, suggesting that exosome biogenesis and secretion are increased after DNA damage [23]. This indicates that radiation-induced DNA damage can affect exosome biogenesis and secretion in a p53-dependent manner. However, these studies cannot prove that radiation can induce exosome biogenesis and increase exosome secretion. Some researchers have conducted further experiments and proved the effect. In irradiated nonhuman primates (NHPs), Amrita K. Cheema found that the number of exosomes per µL of plasma increased significantly by Day 1 post irradiation with 5.8 Gy and Day 14 postirradiation after two doses [24]. In MCF-7 human breast cancer cells, Nasrollah Jabbari [25] found that radiotherapy improved the biogenesis and secretion of exosomes in a dose dependent manner. These two studies all showed that radiation can induce exosome biogenesis and increase exosome secretion, but they have not explained the exact mechanism. In human prostate cancer cells, Lehmann found that radiation therapy has the ability to induce senescence by damaging DNA, which could trigger a p53-dependent augmentation of the biogenesis and secretion of exosomes [26]. An observation [27] in TSAP6/Steap3-null mice indicated that p53 activated by IR-induced DNA damage could increase exosome secretion in a TSAP6-dependent process. In conclusion, these studies are limited to a p53-dependent DNA damage pathway, but help to obtain a new understanding of the influence of radiation therapy on exosome biogenesis and secretion (Table 1). Radiation has various influence on tumor and tumor microenvironment and radiation-induced DNA damage has many pathways except the p53-dependent manner. Further studies need to focus on whole tumor microenvironment and not to be limited by previous studies.

**Table 1:** The influence of radiotherapy on exosome biogenesis, secretion and cargoes

Source of exosomes	Study model	Dose of radiation	Test approach	Isolation method	Effect	References
22RV1 cell line	Human prostate cancer	4 Gy	SDS-PAGE	UC	Radiation induced a p53-dependent augmentation of the biogenesis and secretion of exosomes	[26]
MEFs and BMDCs cell lines	TSAP6 knockout mice	10 Gy	FACS analysis, Glycosylation and dimerization analysis, PCR	UC	Radiation increased exosome secretion in a TSAP6-dependent process	[27]
Not applicable	Rhesus macaques	5.8 Gy or 6.5 Gy	Metabolomic and lipidomic analyses	UC	Radiation increased the number of exosomes	[24]
MCF-7 cell line	Human breast cancer	2, 4, 6, 8, 10 Gy	Western blotting, real-time PCR	UC	CD63 and Alix genes was significantly higher after radiation	[25]
LN18, U87MG, U251cell lines	Glioblastoma	2–8 Gy	Immunoblot analysis, qRT-PCR	UC	CTGF and IGFBP2 were upregulated in radiation-derived exosomes	[28]
FaDu cell line	Human head and neck cancer	2–8 Gy	RNA-seq approach	Differential centrifugation, ultrafiltration and precipitation	MiR-3168 significantly upregulated in exosomes after radiation	[29]
MCF-7 cell line	Human breast cancer	2 Gy	Flow cytometry, western blot	Differential centrifugation	An upregulation of miR-30a and miR-9a accompanied by a downregulation of miR-200b was induced by radiation	[30]
Human pancreatic cancer cell line	Human pancreatic cancer	10 Gy	QRT-PCR	Differential centrifugation	Radiation induced 196 filtered differentially expressed exosomal ircRNAs	[31]

*MEFs*, mouse embryo fibroblasts; *BMDCs*, bone marrow-derived dendritic cells; *UC*, ultracentrifugation; *CTGF*, connective tissue growth factor; *IGFBP2*, insulin-like growth factor binding protein 2

## The influence of radiotherapy on exosome cargoes

To date, exosomes have already been confirmed to contain proteins, nucleic acids (e.g., DNA, mRNA, noncoding RNA, long noncoding RNA) and lipids. Due to their endosomal origin, exosomes contain protein families associated with multivesicular body (MVB) formation, membrane transport and fusion (e.g., Rab GTPases), and a broad array of transmembrane proteins, lipid-anchored membrane proteins, peripherally associated membrane proteins, and soluble proteins of the exosome lumen [7, 8]. In terms of lipid species, exosomes usually have more cholesterol, phosphatidylinositol, ceramide, sphingomyelin, and monosialoganglioside than donor cells [32]. However, the amounts of phosphatidylcholine and lysobisphosphatidic acid are lower [33]. Additionally, major histocompatibility complex (MHC) class I molecules, heat shock proteins and tumor antigens are also found in exosomes [34]. Some studies have shown that radiation can not only affect the biogenesis and secretion of exosomes but also influence the cargo of exosomes [26] (Table 1). The B7-H3 protein, whose high expression provides an extremely reliable marker for the differentiation of indolent from aggressive prostate cancers [35], can be detected in exosomes derived from radiation-induced senescent 22Rv1 cells [26]. Arscott et.al found that connective tissue growth factor (CTGF) and insulin-like growth factor binding protein 2 (IGFBP2) were upregulated in radiation-derived exosomes [28]. Real-time quantitative PCR showed that the CTGF transcript was present at levels approximately two times higher in radiation-derived exosomes than in nonirradiated controls.

Exosomes contain many nucleic acids, such as DNA, miRNA, noncoding RNA (ncRNA) and long noncoding RNA [36–38]. Compared to cellular RNAs containing a great deal of full-length ribosomal RNA (rRNA), exosomal RNAs are generally abundant in ncRNAs, including small nuclear RNAs (snRNAs), miRNAs, transfer RNAs (tRNAs), vault RNAs, and repetitive element RNAs [38–40]. There is an intact RNA sorting mechanism to load RNA into exosomes in the endolysosomal compartment [41]. Gag and Gag-like proteins can coimport their genomic RNA and other RNAs into exosomes [42, 43], while YBX1 plays a vital role in loading small ncRNAs into exosomes [40, 44]. Radiation can also affect exosomal nucleic acids, especially miRNAs [29, 30, 45] (Table 1). It was found that miR-3168, involved in the DNA damage response, was significantly upregulated in exosomes after 2 or 8 Gy radiation in FaDu cells (derived from human head and neck cancer) [29]. Gaines et al. reported that the exosomal levels of hsa-miR-762, has-let-7b-5p and hsa-let-7c-5p, which can regulate the genes associated with cognitive, motor delay and mental, were significantly downregulated after exposure to 3 Gy proton radiation [45]. And further analysis showed that these miRNAs may be biomarkers for neurological radiation injury. There are many studies on exosomes, but this study provides a new direction. Although this study used extracellular vesicles, it still revealed the role of exosomes. Further studies need to focus on the role of exosomes in neurological radiation injury, which may have implications for the treatment of neurological radiation injury rather than merely serving as a biomarker. Abedi et al. reported a significant upregulation of miR-30a, miR-9a and TGF-β Protein accompanied by a significant downregulation of miR-200b after 2 Gy X-ray [30]. Further analysis showed that these miRNAs and protein enhance invasiveness of nonirradiated cells. However, they did not study the exact mechanism. Chen et al. [31] reported 196 filtered differentially expressed exosomal circRNAs in pancreatic cancer cells after radiation. These overexpressed exosomal circRNAs can regulate metabolic process and lysine degradation in tumor cells. And they may involve in pancreatic cancer cell repopulation via the hsa_circ_0002130-hsa_miR_4482-3p-NBN interaction network. In conclusion, these results showed that radiotherapy can influence exosome cargoes and these exosome cargoes can influence the effect of radiotherapy conversely, but further trials are needed to clarify the clinical significance of this interaction and how to turn it into clinical value.

## Radiation-induced exosomes in radioresistance

Radioresistance can be classified into two categories: intrinsic radioresistance and acquired radioresistance [46], but they are all derived from tumor cells’ strong propensity to live during fractionated radiotherapy. Radioresistance comes not only from the protective processes of the tumor stroma and microenvironment but also from genetic or phenotypic changes within the tumor [47, 48]. Recently, many studies have shown that exosomes are involved in radioresistance. For instance, hepatocellular carcinoma (HCC) is considered a radioresistant tumor in clinical and some researches showed that HCC derived exosomes played a vital role in radioresistance [47, 49, 50]. Therefore, studies on the function of radiation-induced exosomes in radioresistance may help to uncover the specific mechanism of radioresisiatnce and find the way to alleviate radioresistance.

Radiation chiefly causes double-strand breaks (DSBs) to induce lethal cytotoxicity and tumor cell apoptosis [3]. Radiation-induced DNA damage can increase exosome biogenesis and exosome secretion in a p53-dependent manner [26, 27]. Interestingly, after treatments with low-dose radiation-induced exosomes, the growth of xenografted tumors was accelerated and the survival period was reduced [51]. It was found that low-dose radiation increased the secretion of exosomes with a high level of circ-METRN in glioblastoma cells. Treated with circ-METRN-abundant exosomes, γ-H2AX (radiation-induced phosphorylation of H2AX, a marker of DNA breaks) was highly expressed in glioblastoma cells, indicating an efficient DNA damage-repair process in glioblastoma cells. Therefore, circ-METRN-abundant exosomes may be involved in radioresistance by inducing high activation of the DNA damage repair process. The specific mechanism may involve the miRNA-4709-3p/GRB14/PDGFRα pathway. Circ-METRN-abundant exosomes were transported into glioblastoma cells and acted as miRNA-4709-3p sponge. Then miRNA-4709-3p targeted GRB14 and affected the expression of GRB14 mRNA and protein. GRB14 plays a glioblastoma-promoting role by regulating the downstream PDGFRα after treatment with low-dose radiation-induced exosomes. This study is the first to reveal the role of exosomal circ-METRN via the miRNA-4709-3p/GRB14/PDGFRα pathway in radioresistance. Studies have shown that radiation can increase exosome biogenesis and exosome secretion by inducing DNA damage. Conversely, radiation-induced exosomes can mediate resistance to radiotherapy by inducing high activation of the DNA damage repair process. These solid research foundations seem to explain the inevitability of radioresistance and suggest the way to alleviate radioresistance. However, the researchers only conducted cell experiments with glioblastoma cells. The generalizability of the results is debatable. In other studies, it was found that exosomes derived from mesenchymal stem cells enhanced radiotherapy-induced cell death in tumor and metastatic tumor foci [52], which is contrary to this result. Previous studies showed that CD44 are associated with radioresistance in prostate cancer [53], glioblastoma [54] and head and neck squamous cell carcinomas [55]. Then, Wang et.al developed exosomes from γδ-T cells and found that γδ-T-exosomes can specifically target the radioresistant CD44^+/high^ CSCs in nasopharyngeal carcinoma. In addition, γδ-T-exosomes combined with radiotherapy had a higher therapeutic efficacy than radiotherapy monotherapy in vitro and in vivo [56].

This study has some limitations but still shows a powerful preclinical evidence using exosomes to alleviate radioresistance.

At present, it has been confirmed that exosomal miRNAs are associated with radioresistance [57–64] (Table 2). In lung cancer cells [57], exosomal miR-208a was significantly increased after 60 Gy X-ray. And miRNA-208a decreased cellular apoptosis and disturbed the cell cycle by targeting p21 with a corresponding activation of the AKT/mTOR pathway, which ultimately promotes cell proliferation and induces radioresistance. Chen et al. reported that exosomal miR-93-5p from cancer-associated fibroblasts conferred radioresistance in colorectal cancer cells by downregulating FOXA1 and upregulating TGFB3 [60]. They also reported that exosomal miR-590-3p has the same function via the positive regulation of the CLCA4-dependent PI3K/Akt signaling pathway [61]. These three studies showed that exosomal miRNAs involved in radioresistance and may be potential therapeutic target to alleviate radioresistance though the mechanism needed further research. Wan et.al found that microRNA-34c-5p (miR-34c) inhibited malignant behaviors in nasopharyngeal carcinomas [59]. Exosomes derived from miR-34c-transfected mesenchymal stem cells (MSCs) attenuated nasopharyngeal carcinoma invasion, migration and proliferation. Furthermore, miR-34c-overexpressing exosomes significantly increased radiation-induced apoptosis in nasopharyngeal carcinomas. This study showed that exosomal miRNAs can enhance radiotherapy, which is contrary to the study in lung cancer cells. Therefore, exosomal miRNAs have various function in radiotherapy and further studies need to focus on more miRNAs and the mechanism.

**Table 2:** Radiation-induced exosomes in radioresistance

Source of exosomes	Study model	Dose of radiation	Isolation method	Effect	References
SW1783 and U-118MG cell lines	Glioblastoma	2, 4, 6, 8 and 10 Gy	Differential centrifugation	Exosomal circ-METRN involved in radioresistance via the miRNA-4709-3p/GRB14/PDGFRα pathway	[51]
A549, H1299, H1975 and H460 cell lines	Human lung cancer	60 Gy	Differential centrifugation	Exosomes can transmit miRNA-208a to induce radioresistance	[57]
SW1990 cell line	Human pancreatic cancer	10 Gy	Not applicable	Exosomal miRNA-194-5p potentiated tumor repopulation by enhancing the DNA damage response	[58]
Cancer-associated fibroblasts and normal fibroblasts cell lines	Human colorectal cancer	6 Gy	Ultracentrifugation	Exosomal miR-93-5p induced radioresistance by downregulating FOXA1 and upregulating TGFB3	[60]
Cancer-associated fibroblasts and normal fibroblasts cell lines	Human colorectal cancer	12 Gy	Differential centrifugation	Exosomal miR-590-3p induced radioresistance by the positive regulation of the CLCA4-dependent PI3K/Akt signaling pathway	[61]
Whole blood from oesophageal squamous cell carcinoma patients	Human oesophageal squamous cell carcinoma	8 Gy	Differential centrifugation	Exosomal miR-340-5p promoted radioresistance of oesophageal squamous cell carcinoma via KLF10	[62]
KYSE-150 and TE-1 cell lines	Human esophageal squamous carcinoma	8 Gy	Not applicable	The exosomal transfer of miR-199a-5p involved in radioresistance	[63]
LIM1863 cell line	Human colorectal cancer	68 Gy	Differential centrifugation	Exosomal microRNA-19b targets FBXW7 to induce radioresistance	[64]

## Radiation-induced exosomes in RIBEs

Radiotherapy can induce radiation effects in nonirradiated cells and tissues by intercellular communication, which is named radiation-induced bystander effects (RIBEs). RIBEs include genomic instability, DNA damage, stress responses, senescence, cell apoptosis and proliferation [4, 65–67]. Compared with the direct effects of radiation, RIBEs play a crucial role in the low-dose range [68–70] but not in the high-dose range [70, 71]. RIBEs tend to work 1–5 mm away from directly irradiated cells [72–74].

First, the signals from directly irradiated cells transmitted into nonirradiated contact neighboring cells by gap junction intercellular communication [75, 76]. Then, soluble signals such as reactive oxygen species (ROS) or secreted factors such as cytokines [77, 78] trigger RIBEs between the targeted cells and the distanced nontargeted cells by medium communication.

Recent studies showed that exosomes can play a crucial role in RIBE [6, 23, 52, 79–83] (Table 3). Exosomes can even deliver genomic instability from irradiated cells to bystander cells [30].

**Table 3:** RIBEs caused by exosomes

Donor cells	Recipient cells	Dose of radiation	Isolation method	Effect	References
Human non–small cell lung cancer cell lines (H460, H1299)	H460, H1299	5 Gy	UC	Exosomes communicated with adjacent cells	[23]
Human breast cancer cell line (MCF-7)	MCF-7	2 Gy	UC	Exosomes were partially involved in genomic instability	[79]
Human breast cancer cell line (MCF-7)	MCF-7	2 Gy	UC	Exosomal RNA and protein molecules were associated with RIBEs	[80]
Human embryonic lung fibroblasts (MRC-5)	MRC-5	2 Gy	UC	Exosomal miRNA-21 induced DNA damage and chromosome aberrations in bystander cells	[6]
Human papillomavirus– immortalized human bronchial epithelial (BEP2D),	BEP2D	2 Gy	UC	Exosomal miR-7-5p mediated bystander autophagy	[86]
Human HNSCC cell lines (BHY, FaDu)	BHY, FaDu	0–9 Gy	UC	Exosomes promoted the proliferation and radioresistance	[83]
Human papillomavirus– immortalized human bronchial epithelial (BEP2D),	BEP2D	2 Gy	UC	Exosomal microRNAs contributed to DNA damage	[82]
Human umbilical-cord stromal stem cells (MSCs)	Human melanoma cell lines (A375, G361) and human breast cancer cell line (MCF-7)	2 Gy	UC	Exosomes enhanced bystander tumor growth and metastasis	[52]
BALB/C mouse–derived mammary carcinoma (TSA)	DCs	8 Gy	UC	Exosomes transferred anti-tumor effects to bystander cells	[81]
Human breast cancer cell line (MCF-7)	MCF-7	2 Gy	UC	Exosomes enhanced invasiveness of bystander cells	[30]

*HNSCC*, head and neck squamous cell carcinoma; *DCs*, dendritic cells; *UC*, ultracentrifugation

Abedi et al. introduced CCCM (control cells conditioned media) and ICCM (irradiated cells conditioned media) onto unirradiated MCF-7 cells and assessed the cell invasion by evaluating the number of invaded cells [30]. Compared with MCF-7 cells incubated with CCCM, those incubated with ICCM had a higher number of invaded cells. Further study showed that MCF-7 cells incubated with ICCM-derived exosomes had an increased invasive potential as those incubated with ICCM. This indicated that exosomes involved in RIBE via delivering genomic instability from irradiated cells to bystander cells. In addition, exosomes can also deliver cell apoptosis from irradiated cells to bystander cells. Combining MSC cell therapy and radiotherapy in melanoma tumor xenografts implanted in NOD/SCID-gamma-mice, the size of the established tumors, both in the primary-directly irradiated tumor and in the distant nonirradiated tumor, was reduced [52]. Then Farias et al. compared the survival fractions of A375 cells treated with irradiated MSC conditioned medium or irradiated MSC exosomes. Studies showed that exosomes from irradiated MSC reduced the cell survival of A375 cells the same as the irradiated MSC conditioned medium [52]. These studies showed that exosomes can deliver genomic instability and cells apoptosis from irradiated cells to bystander cells, but the mechanism is not clear.

Exosomes can transport miRNA from irradiated cells to nonirradiated cells, which play a vital role in RIBEs [5, 6]. MiRNA-21 in both directly irradiated cells and bystander cells was significantly upregulated via identification of a set of differentially expressed microRNAs in the human fetal lung MRC-5 fibroblast (human embryonic lung fibroblast) culture medium after irradiation [84]. Transfection of miRNA-21 mimics into nonirradiated MRC-5 cells caused an apparent increase in the frequency of micronuclei and 53BP1 foci and a dramatic decrease in the survival fraction, suggesting that miRNA-21 is involved in RIBEs. Further research found that exosomes can transfer miRNA-21 from irradiated cells into the extracellular medium and subsequently obtain access to the recipient cells to induce DNA damage and chromosome aberrations [6]. These studies showed that miRNA-21 involved in RIBEs and may be a potential target to upregulate RIBEs to kill nonirradiated tumor cells.

Wang et al. reported that the expression of the autophagy markers LC3-II/LC3-I and Beclin-1 increased in bystander HepG2 (human hepatocellular carcinoma) cells treated with conditioned medium (CM) collected from irradiated HepG2 cells [85]. They found that the transfection of LC3 siRNA or Beclin-1 siRNA significantly enhanced the yield of micronuclei in bystander cells. Therefore, autophagy may also play a role in modulating the bystander effects. Song et al. used the human bronchial epithelial cell line BEP2D (bronchial epithelial cells) as the normal cellular model for further study [86]. It was found that the recipient BEP2D cells took in more miRNA-7-5p-abundant exosomes from the IR-irradiated cells compared with nonirradiated cells by labeling the exosomes from the conditioned medium of 2 Gy irradiated cells with CM-Dil fluorescent dye. Then miRNA-7-5p targeted EGFR (epidermal growth factor receptor) and decreased its expression, which was largely attenuated by a miRNA-7-5p inhibitor. Next the phosphorylation levels of phospho-Akt and phospho-mTOR decreased and subsequently regulated autophagy progression. Therefore, exosomes can transfer miRNA-7-5p from irradiated cells to nonirradiated cells to induce RIBE via the EGFR/Akt/mTOR signaling axis. This study showed that exosomal miRNA involved in RIBEs with exact mechanism and miRNA-7-5p may be potential target to downregulate RIBEs to protect normal cells.

Radiotherapy can also induce various dysregulated proteins and nucleic acids. These substances invaginate the LSE and are then transported to nonirradiated cells by exosomes, which also cause RIBEs [87, 88]. In a mouse model, exosomes derived from irradiated mouse breast cancer cells could transfer dsDNA to DCs and stimulate the upregulation of costimulatory molecules in DCs, suggesting that exosomes derived from irradiated cells could transfer antitumor effects to nonirradiated cells [81]. These studies indicated that further studies should focus on all exosome cargoes.

## Discussion

Radiotherapy, as a mainstay treatment for cancer, is used in more than half of cancer patients to cure localized cancer, palliate symptoms, or control the progression of cancer. However, radioresistance is still the main reason for the failure of radiotherapy. It needs a biomarker that can predict the efficacy of radiotherapy to assist in rapid clinical adjustment of treatment plans through real-time monitoring [89]. Radiation can affect the biogenesis, cargo and secretion of exosomes. Conversely, exosomes can mediate radioresistance, and change the expression levels of their cargos which closely related to treatment response. These findings suggest that exosomes may be invasive, novel and sensitive biomarkers for monitoring the efficacy of radiotherapy. However, the interaction between radiotherapy and exosomes is complex, and it is difficult to quantify one or more factors into an index to evaluate radioresistance. The clinical value and practicability of exosomes as biomarkers also need to be evaluated.

Exosomes as natural nanoscale vesicles have attractive advantages in cancer treatment due to their high biological permeability, high biocompatibility, and low immunogenicity [90–93]. Besides, double-layer lipid and high stability of exosomes enable exosomes to maintain biological activity for a longer time in blood circulation even after repeated manipulations [91]. Therefore, exosomes may be used as delivery systems for therapeutic loads such as RNA (mRNA/miRNA and other non-coding RNA/interfering small RNA) and chemotherapeutic drugs and immunomodulators. However, it needs a method that can efficiently and massively load drugs onto exosomes, which is a prerequisite for exosomes to be used as delivery Systems. Additionally, exosomes derived from diverse cells have different biological functions. Research on exosomes-based cell-specific drug delivery needs to be verified. Without the ability to deliver and release these drugs into the tumor microenvironment, precision therapy is just a theory. Recently, Wang et.al first reported the application of exosomes for anaplastic thyroid carcinoma [94]. They engineered HEK-293 T (the human embryonic kidney epithelial cell line) cells to developed an exosomes-based targeted delivery platform loading with doxorubicin (Dox) and labeled with radioiodine-131 (^131^I). This vehicle specifically targeted to tumor and inhibited the growth of tumor with biosafety and no side effects by intravenous injection to a mouse model. This is a great advance in exosomes research, which proves the feasibility of exosomes as a delivery system to transport drugs and brings a lot of inspiration for future in vivo trials. However, this is the only one report on this aspect, and more experiments are needed to verify its authenticity and universality.

Radiation-induced exosomes involved in radioresistance by specific mechanism. The presence of radioresistant exosomes may be a signal to intensify treatment by radiation-enhancing agents, engage in radiotherapy dose escalation, or stop irradiation. In the meantime, exosomes may be a therapeutic target to alleviate radioresistance and increase radiosensitivity, which will improve the therapeutic effectiveness of radiotherapy. Exosomes are not only involved in radioresistance but are also involved in radiation-induced bystander effects (RIBEs) mediated by miRNAs. Therefore, the risk of unirradiated normal tissue toxicity could be evaluated by exploring exosomal miRNAs. This evaluation could provide clinical guidance to use more stringent normalization tissue dose constraints or to avoid radiotherapy. Exosomes may also be a therapeutic target to modulate RIBEs to kill cancer cells on the basis of protecting normal cells as much as possible in nonirradiated tissue. While these ideas have great potential clinical value, research in these areas is scarce and at a basic level. More experiments are needed to understand the mechanisms involved and use them to create clinical value.

Radiation can kill normal cells as well as tumor cells in clinical target volume. Clinically, there are three main ways to protect normal cells from radiation: radioprotective agents delivered before radiation exposure, after radiation exposure and after the onset of symptoms [95]. Preclinical studies showed that amifostine has a good radioprotective effect [96], but severely limited due to its severe adverse effects and short half-life [97, 98]. As traditional radioprotectants cannot meet the clinical need, it is crucial to find a new radioprotectors. Some studies showed that exosomes play a vital role in treating and preventing radiation injury such as skin injury and bone loss [99, 100]. The major cause of skin injury induced by radiation is oxidative stress. In irradiated mice skin, MSC-derived exosomes treatment reduced reactive oxygen species generation and improved antioxidant capacities via adaptive regulation of the NRF2 defense system [99]. In the other rat model, exosomes derived from bone marrow mesenchymal stem cells (BM-MSCs) reduced oxidative stress and proliferation inhibition and accelerated DNA damage repair after irradiation [100]. Exosomes facilitate β-catenin expression and restore the balance between osteogenic differentiation and adipogenic in irradiated BM-MSCs. These experiments illustrate the role of exosomes in reducing radiation injury and provide a new treatment for radiation injury in clinical. Although there are few studies in this field, they have shown great promise of exosomes in reducing radiation injury.

Exosomes have many advantages, such as high biocompatibility, long life, and low immunogenicity [101–103]. Yet, limited understanding of biogenesis, cargoes, secretion and target cell uptake of exosomes has greatly restricted investigation on the role of exosomes in radiotherapy [89, 104]. First, exosomes from diverse cells have different potential biological functions. Therefore, exosomes need to be targeted as the cell-specific delivery vehicle for therapy. Second, many factors, such as irradiation dose and the pH value of the culture medium can affect the process of cargoes loading into exosomes [29, 105] and there no unified standards for purification and quantification of exosomes. Third, there is a lack of how to effectively load exogenous ncRNAs or drugs into exosomes. Fourth, the immune responses are also unclear when utilizing non-autologous exosomes. Last, it is needed to prolong the half-life of exosomes in vivo to maintain a high blood concentration.

Unlike normal tissues, highly aggressive, rapidly growing solid tumors encounter hypoxia as a result of fluctuating and/or inadequate a blood supply [106]. Tumor hypoxia drives the tumor toward a more malignant phenotype by stimulating the invasion of tumor cells, induces radioresistance and is an adverse clinical prognostic factor [107–110]. By measuring the partial pressure of oxygen (pO_2_) in head and neck carcinomas, we found a correlation between low pO_2_ and poor local control or survival after radiotherapy [111]. Therefore, hypoxia needs to be taken into consideration to determine whether exosomes can be applied to clinical. Studies on exosome functions in radiotherapy are in their infancy and need further in vivo experiments.

## Conclusions

Radiotherapy remains the fundamental therapy for tumors, but it is not perfect and it currently cannot meet requirements to maintain high local control rates (LCRs) and overall survival rates (ORs). Radiotherapy not only induces radioresistance but also causes bystander effects leading to radiation injury. Therefore, it is necessary to explore the specific mechanism of radioresistance and RIBEs. Despite functional and methodological challenges, the investigation of exosomes could help to unveil the mechanisms of radioresistance and RIBEs. Additionally, detailed mechanisms underlying the crosstalk between exosomes and radioresistance and RIBEs are being discovered by recent studies. We found that tumor-derived exosomes could protect tumor cells from radiation. Exosomes can also transport cargos from irradiated cells to nonirradiated cells to induce RIBEs. Although the relevant research is still in the embryonic stage, it still shows the great potential clinical value of exosomes in radiotherapy. Radioresistant exosomes may be an indicator of the prognosis of radiotherapy patients due to the presence of them will reduce the efficacy of radiotherapy. Radioresistant exosomes may also be a therapeutic target to alleviate radioresistance and increase radiosensitivity. Recent studies showed the crucial role of exosomes in RIBE. This indicated that we may regulate RIBE via intervening exosomes to kill tumor cells and protect normal cells at the same time. We also found that exosomes could be used to alleviate radiation injury such as skin injury and bone loss. However, RIBE is a double-edged sword and exosomes can facilitate both of them. It is difficult to achieve the win–win idea of trying to regulate RIBE with exosomes. In conclusion, exosomes involve in various mechanisms of radiotherapy and show great potential clinical value. Exosomes could be an indispensable combination therapy with radiotherapy. More experiments are need to study the specific mechanism of exosomes in radiotherapy and how to apply it in clinical.

## Data Availability

Not applicable.

## Abbreviations

BEP2D: Bronchial epithelial cells
CM: Conditioned medium
CSC: Cancer stem-like cell
CTGF: Connective tissue growth factor
DSBs: Double-strand breaks
EGFR: Epidermal growth factor receptor
EMT: Epithelial-mesenchymal transition
ESE: Early-sorting endosome
ESCRT: Endosomal sorting complex required for transport
EVs: Extracellular vesicle
HCC: Hepatocellular carcinoma
HepG2: Human hepatocellular carcinomas
HEK-293 T: The human embryonic kidney epithelial cell line
IGFBP2: Insulin-like growth factor binding protein 2
ILVs: The formation of intraluminal vesicles
LCRs: Local control rates
lncRNA: Long noncoding RNA
LSE: Late-sorting endosome
MHC: Major histocompatibility complex
miRNAs: MicroRNAs
MRC-5 fibroblasts: Human embryonic lung fibroblasts
MSCs: Mesenchymal stem cells
MVBs: Multivesicular bodies
ncRNA: Noncoding RNA
NHPs: Nonhuman primates
ORs: Overall survival rates
PGE2: Prostaglandin E2
RIBEs: Radiation-induced bystander effects
rRNA: Ribosomal RNA
RLC: RISC-loading complex
ROS: Reactive oxygen species
SNAP-23: Synaptosome-associated protein 23
SNAREs: Soluble N-ethylmaleimide-sensitive fusion attachment protein receptors
snRNAs: Small nuclear RNAs
SSBs: Single-strand breaks
TRCs: Tumor repopulating cells
tRNAs: Transfer RNAs
VEGF: Vascular endothelial growth factor
VEGF-R1: Vascular endothelial growth factor-receptor 1
YBX1: Y-box binding protein 1
